# Isolation, Expansion, and Characterization of Rat Hair Follicle Stem Cells and Their Secretome: Insights into Wound Healing Potential

**DOI:** 10.3390/biomedicines12122854

**Published:** 2024-12-15

**Authors:** Patrícia Sousa, Bruna Lopes, Ana Catarina Sousa, André Coelho, Alícia de Sousa Moreira, Alexandra Rêma, Maria Gonçalves-Maia, Irina Amorim, Rui Alvites, Nuno Alves, Stefano Geuna, Ana Colette Maurício

**Affiliations:** 1 Departamento de Clínicas Veterinárias, Instituto de Ciências Biomédicas de Abel Salazar (ICBAS), Universidade do Porto (UP), Rua de Jorge Viterbo Ferreira, No. 228, 4050-313 Porto, Portugal; pfrfs_10@hotmail.com (P.S.); brunisabel95@gmail.com (B.L.); anacatarinasoaressousa@hotmail.com (A.C.S.); andrefmc17@gmail.com (A.C.); alicia.moreira.1998@gmail.com (A.d.S.M.); alexandra.rema@gmail.com (A.R.); ruialvites@hotmail.com (R.A.); 2 Centro de Estudos de Ciência Animal (CECA), Instituto de Ciências, Tecnologias e Agroambiente da Universidade do Porto (ICETA), Rua D. Manuel II, Apartado 55142, 4051-401 Porto, Portugal; m-maia@coriumbiotech.com; 3 Associate Laboratory for Animal and Veterinary Science (AL4AnimalS), 1300-477 Lisboa, Portugal; 4 Maia & Muller-Biotech, Rua Alfredo Allen, 455/461, 4200-135 Porto, Portugal; 5 Departamento de Patologia e Imunologia Molecular, ICBAS—School of Medicine and Biomedical Sciences, University of Porto (UP), Rua de Jorge Viterbo Ferreira 228, 4050-313 Porto, Portugal; iamorim@ipatimup.pt; 6 Institute for Research and Innovation in Health (i3S), Universidade do Porto, Rua Alfredo Allen 208, 4200-135 Porto, Portugal; 7 Institute of Molecular Pathology and Immunology, University of Porto (IPATIMUP), Rua Júlio Amaral de Carvalho 45, 4200-135 Porto, Portugal; 8 Instituto Universitário de Ciências da Saúde (CESPU), Avenida Central de Gandra 1317, 4585-116 Paredes, Portugal; 9 Centre for Rapid and Sustainable Product Development, Polytechnic of Leiria, 2430-028 Marinha Grande, Portugal; nuno.alves@ipleiria.pt; 10 Department of Clinical and Biological Sciences, Cavalieri Ottolenghi Neuroscience Institute, University of Turin, Ospedale San Luigi, 10043 Turin, Italy; stefano.geuna@unito.it

**Keywords:** hair follicle stem cells, regenerative medicine, stem cell characterization, stem cell isolation, wound healing

## Abstract

**Background:** Stem cells are capable of self-renewal and differentiation into various specialized cells, making them a potential therapeutic option in regenerative medicine. This study establishes a comprehensive methodology for isolating, culturing, and characterizing rat hair follicle stem cells. **Methods and Results:** Hair follicles were harvested from Sprague–Dawley rats and subjected to two different isolation techniques. Immunohistochemical analysis and real-time PCR confirm the expression of specific surface markers and genes, validating the cells’ identity. Growth kinetics, colony formation units (CFU), and tri-differentiation capacity were also assessed. Additionally, the cells’ secretome was analyzed, regarding its content in biofactors with wound healing properties. **Conclusions:** These findings highlight the potential of these cells as a valuable cell source for skin regeneration applications. They contribute to advancing our understanding of stem cell applications in regenerative medicine and hold promise for therapeutic interventions in various clinical contexts, aligning with broader research on the diverse capabilities of hair follicle stem cells.

## 1. Introduction

The skin, the body’s largest organ, performs numerous essential functions, acting as a protective barrier against dehydration, a sensory and thermoregulatory organ, and a site for vitamin D synthesis and immune defense. It comprises the epidermis, morphologically organized into layers reflecting the terminal differentiation of keratinocytes, and the dermis [1,2,3].

Skin wound healing is a highly organized process responsible for the restoration of tissue integrity and function. However, disruptions in this process can lead to the development of nonhealing chronic wounds. Various factors and conditions, such as vascular issues, diabetes, trauma, advanced age, and local pressure effects, can delay wound healing. Additional local factors, like tissue hypoxia, infection, and disrupted inflammatory responses, along with systemic factors, like compromised nutritional or immune status, can also impair the healing process. The increasing prevalence of concomitant diseases, such as diabetes and vascular complications, contributes to the global challenge of chronic wound healing, with significant management costs [1,4].

In adult wound healing, the predominant forms of repair involve fibrosis and scar formation, instead of regeneration. A coordinated interplay of various cell types, growth factors, and cytokines is essential for wound closure [5].

In recent years, regenerative medicine has gained increasing significance as an alternative to conventional therapies in several different diseases lacking effective treatment. There has been an increased effort to identify alternative therapeutic sources, such as stem cells and their derivates, which are easily accessible, safe, and stable and have great pro-regenerative potential [6,7].

The skin’s repair and regenerative capabilities are closely tied to the presence of stem cells. Currently, there is no consensus on the specific type, density, and function of skin stem cells. Nevertheless, it has been demonstrated that skin stem cells, particularly hair follicle stem cells (HFSCs) located in the hair follicle bulge, may serve as crucial sources for skin regeneration, metabolism, and wound repair [8,9]. Stem cells are crucial for the maintenance and regeneration of both the epidermis and hair follicles, with epidermal stem cells demonstrating the ability to sustain tissue homeostasis, self-renew, and contribute to wound repair [1].

HFSCs not only contribute to the development of hair follicles but also accelerate wound healing. In general, a higher abundance of residual skin stem cells on a wound surface is associated with faster healing and reduced scar formation. For instance, the scalp is often utilized as a skin donor site in clinical practice due to its substantial reservoir of stem cells capable of regeneration and repair. However, in deep wounds, scar hyperplasia may occur after healing, as most skin stem cells are lost [8,9].

The skin harbors various stem cells with multilineage differentiation potentials. Theoretically, skin stem cells can facilitate physiological healing in any wound. Yet, in deep wounds affecting skin appendages, the remaining skin stem cells may struggle to undergo normal differentiation, hindering the completion of anatomical structure and functional skin repair. This may result in uncontrolled healing processes, ultimately forming scar tissues without essential components, such as hair and sweat glands. The outcome of wound healing is not solely determined by the number of skin stem cells but also by their differentiation behaviors [8,9].

Stem cells have demonstrated significant efficacy in animal experiments and clinical studies for various diseases, due to their multilineage differentiation potentials, anti-inflammatory properties, paracrine functions, and other biological capabilities [10,11,12,13,14]. In recent years, the application of different stem cell types in treating wounds has gained recognition. In both systemic and local implantation in animals with refractory wounds, stem cells have shown the potential to differentiate into keratinocytes, sebaceous glands, and other skin appendages, contributing to wound healing. Despite these therapeutic effects, the precise mechanism of action of epidermal stem cells in wound healing remains poorly understood [8].

**Figure 1:**
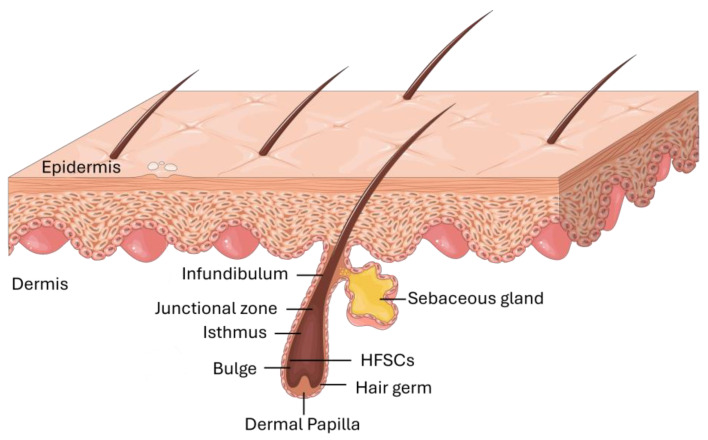
Schematic representation of the HFSCs’ location in the skin.

Most adult mammalian skin is covered with hair, and within the hair follicles are specialized cells known as HFSCs. Located in the bulge region of the outer root sheath, these cells serve as a reservoir, generating transient amplifying cells that are crucial for producing diverse cell types during hair follicle regeneration (Figure 1). Additionally, these versatile stem cells play a role in repairing the epidermis during the process of wound healing [15].

They have recently garnered attention as a promising stem cell source for regenerative medicine due to their ready availability and favorable tissue location. These cells have shown cloning capacity and regenerative potential in vitro, as well as the ability not only to differentiate into hair follicles but also into various cell types, including nerve cells, smooth muscle cells, epithelial cells, sebaceous glands, sweat glands, and epidermis. Abundantly present in the skin and hair follicles, autologous HFSCs can be easily isolated from patients, and the harvesting process has not been associated with serious complications [16,17,18].

HFSCs offer several advantages, such as easy accessibility, simple culture and expansion processes, the absence of MHC class I expression (reducing the likelihood of graft rejection), high proliferative capacity, multipotential properties, and the potential for autologous use without side effects. Furthermore, HFSCs share a common ectodermal embryonic origin with skin keratinocytes, making them potentially more effective in cell therapy for cutaneous wound healing [19].

The use of secretome therapies, harnessing the diverse range of bioactive molecules produced and secreted by cells, offers significant advantages in wound healing by modulating inflammation, tissue repair, and remodeling [20,21,22,23].

Irons et al. demonstrated that adipose-derived stem cells, endothelial-differentiated adipose-derived stem cells, and their secretomes significantly improved diabetic skin regeneration in a swine model by promoting angiogenesis and reducing inflammation [24].

Heo et al. showed that TNF-α-activated adipose-derived stem cells’ secretome accelerated wound closure, enhanced angiogenesis, promoted cell proliferation, and increased the infiltration of immune cells into cutaneous wounds in a rat excisional model [25].

Suzdaltseva et al. demonstrated the efficacy of umbilical cord-derived mesenchymal stem cells in a human clinical trial of chronic wound regeneration, with improvements in granulation tissue growth, blood microcirculation, and wound size reduction [26].

These molecules collectively contribute to the effectiveness of secretome-based therapies in promoting wound healing and tissue regeneration [22,27,28].

The aim of this study was to provide a detailed description of the techniques used in the isolation, culture, expansion, cryopreservation, and thawing processes of rat-derived HFSCs. In addition, cell characterization was conducted, encompassing cell behavior in culture, the exploration of cell genetic expression, the identification of specific cell surface proteins, the assessment of multilineage differentiation capacity, colony formation ability, cytogenetic analysis, and secretome characterization. For the first time, a comprehensive methodology was established for these processes in rat HFSCs (rHFSCs).

While various protocols for isolating rHFSCs have previously been developed and described, the authors introduced a novel approach utilizing an L929 feeding layer, a method that had not been previously explored. To further refine this process, two distinct protocols were compared to identify the most effective strategy. Additionally, this study addressed a significant gap in the field by conducting a detailed characterization of the rHFSC secretome, which had been largely overlooked in prior research, thereby providing critical insights into the therapeutic potential of these cells.

These findings contribute to advancing the understanding of stem cells in regenerative medicine, offering potential therapeutic interventions, specifically in skin regeneration and wound healing. Moreover, these results align with broader research exploring the diverse capabilities inherent in hair follicle stem cells and their potential in regenerative medicine.

## 2. Materials and Methods

All experiments involving animals were conducted in strict accordance with ethical guidelines and were granted approval by the Organism Responsible for Animal Welfare (ORBEA) at the Abel Salazar Institute for Biomedical Sciences (ICBAS) of the University of Porto (UP). Additionally, approval was obtained from the Veterinary Authorities of Portugal (DGAV). All animal procedures adhered to the principles outlined in Directive 2010/63/EU of the European Parliament and the Portuguese DL 113/2013. The study also followed the recommendations set forth in the OECD Guidance Document on the Recognition, Assessment, and Use of Clinical Signs as Humane Endpoints for Experimental Animals Used in Safety Evaluation (2000).

Four male Sprague–Dawley rats (Charles River, Barcelona, Spain), aged 8–10 weeks, with a body weight ranging from 200–300 g, were used for obtaining the sample tissue. In line with the 3 Rs principle (replacement, reduction, and refinement), and in collaboration with other research endeavors within the group, animals that were sacrificed in the context of other assays were reused to collect vibrissae, and no animals were sacrificed specifically for this work. The trials in which these animals participated did not directly or indirectly affect the vibrissae region. Animals were pre-anesthetized with the intraperitoneal administration of xylazine (Rompun^®^, Greenfield, IN, USA, 1.25 mg/g) and ketamine (Imalgene 1000^®^, Rhein, Germany, 9 mg/100 g) in a single administration. Euthanasia was carried out using a chemical method, involving an anesthetic overdose with pentobarbital sodium (Eutasil^®^ 200 mg/mL injectable solution, Ceva Santé Animale, Libourne, France, 200 mg/kg) administered intraperitoneally. This approach was undertaken to minimize pain and discomfort, aligning with humane endpoints for mitigating animal suffering and distress.

### 2.1. Mitomycin C Concentration Determination Assay with L929 Cells

The L929 cells were seeded in 12-well plates at 6000 cells/cm^2^ to ensure 70–80% confluency by the time of the assay (18,000 cells/cm^2^). The mitomycin C stock solution was prepared by dissolving mitomycin C powder (10107409001, Roche^®^, Basel, Switzerland) in sterile, distilled water (0.5 mg/mL). Then, the stock solution was diluted to create working solutions with concentrations of 10 µg/mL, 20 µg/mL, 30 µg/mL, and 40 µg/mL in the cell culture medium. The culture medium was removed from each well, and fresh medium containing the desired mitomycin C concentration (10 µg/mL, 20 µg/mL, 30 µg/mL, or 40 µg/mL) was added to the respective wells. Then, the cells were incubated at 37 °C for 3 h (Timepoint 1) and 24 h (Timepoint 2). At Timepoint 1 (3 h post-treatment) and Timepoint 2 (24 h post-treatment), the cell viability and cell number were determined using trypan blue staining. For this, the cells were trypsinized, collected, and stained with trypan blue (0.4% solution) to differentiate viable (unstained) and nonviable (stained) cells. The cell number was counted using a hemocytometer, with three independent counts performed for each condition.

### 2.2. Preparation of the Complete Culture Medium

The complete culture medium was meticulously prepared within a sterile laminar flow hood. Initially, DMEM containing 4.5 g/L of glucose (10566-016 500 mL, Gibco^®^, Waltham, MA, USA) was blended with DMEM-F12 (11039-021 500 mL, Gibco^®^), achieving a proportion of 3:4 for DMEM and 1:4 for Ham’s F12. Subsequently, the following components were sequentially integrated into the medium, with thorough mixing after each addition: 0.1% T3 trio-iodothyronin (T5516—1 mg, Sigma Aldrich^®^, St. Louis, MO, USA), 0.1% adenine (A9795—1 g, Merck^®^, Rahway, NJ, USA), 0.1% transferrin (616395—100 mg, Merck^®^), 0.1% hydrocortisone (386698—25 mg, Merck^®^), 0.1% insulin from bovine pancreas (I5500—50 mg, Sigma Aldrich^®^), and 0.1% cholera toxin (C8052—5 mg, Merck^®^). Additionally, 10% fetal bovine serum—FBS (A4736301, Gibco^®^), 1% L-glutamine (25030-024 100 mL, Gibco^®^), 1% MEM minimum essential amino acids (11140-035, Gibco^®^), 1% sodium pyruvate (11360-039, Gibco^®^), 1% Penicillin-Streptomycin (15140122, Gibco^®^), and 0.2% Amphotericin B (15290026, Gibco^®^) were incorporated. Ultimately, the medium underwent sterilization via a 0.22-micron filter and was stored at 4 °C. The epidermal growth factor recombinant protein (EGF) 0.05mg (91-020, ProSei^®^, Poway, CA, USA) was introduced into the medium 24–48 h after cell seeding to facilitate optimal growth. Moreover, a separate portion of complete culture medium was prepared with the inclusion of 10% Penicillin-Streptomycin to effectively mitigate the risk of sample contamination during isolation.

### 2.3. Collection of Rat Vibrissae-Containing Skin Samples and Transportation

The skin of the rostrum region, including the vibrissae and the respective hair follicle, were collected from Sprague–Dawley rats using a combination of tweezers and a scalpel. These samples were immediately placed in a 10 mL complete medium with 10% Penicillin-Streptomycin and transported on ice to ensure optimal conditions during transit. The samples were processed upon arrival at the laboratory.

### 2.4. Isolation of rHFSCs

Two methods were assessed to identify the superior approach for rHFSC isolation.

In the first method (Method A), the mitomycin C-DMEM solution was introduced into the pre-established L929 feeding layer and incubated for a minimum of 3 h before cell isolation.

In the second method (Method B), the mitomycin C-DMEM solution was incorporated into the pre-established L929 feeding layer, and after a minimum 3-h incubation, complete medium was left conditioning for 3 days before the isolation process.

Immediately after arrival, the skin samples were washed twice in 30 mL of 70% alcohol solution, followed by two washes in 30 mL of PBS solutions (1-min each, manually agitated).

**Figure 2:**
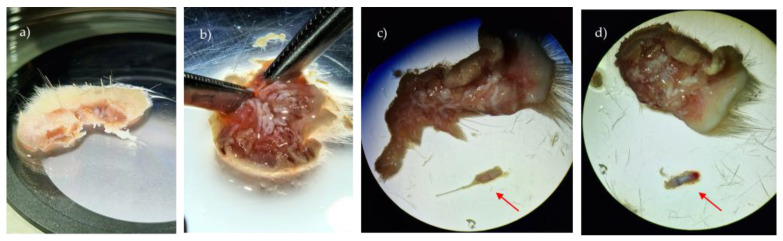
Sprague–Dawley skin samples containing hair follicles, observed under the stereomicroscope (zoom range: 0.63–8×): (**a**) skin sample after collection; (**b**) elimination of adipose tissue and connective tissue; (**c**) observation of follicles integrated into the basal layer and already individualized (arrow); and (**d**) completed extracted hair follicle (arrow).

Using a stereomicroscope (SMZ1270, Nikon^®^, Tokyo, Japan), subcutaneous fat and connective tissue were removed to expose the folliculum in a petri dish (Figure 2), maintaining tissue wetness with PBS solution, as needed. The individual extraction of the folliculum using a scalpel and forceps followed, to gently lift the follicles from the dermis. The base of each follicle was gripped with minimal force to avoid damage, and the process was performed slowly to maintain follicle integrity. Throughout the procedure, tissues were kept hydrated with PBS, containing 2% Penicillin-Streptomycin to prevent desiccation, ensuring the preservation of the natural structure and viability of the hair follicles. The folliculi were centrifuged at 1600× rpm for 5 min, and the supernatant was discarded.

The folliculi were then placed in a falcon with 2 mL of 1% collagenase type IV (17104-019 1 g, Gibco^®^) and incubated at 37 °C with 5% CO_2_ for 1 h. Afterward, the falcon was centrifuged at 1600× *g* rpm for 5 min, and the supernatant was removed. The folliculi were sectioned into small fragments, and the digested tissue fragments were transferred into a 12.5 cm^2^ T-flask, with 5 mL dispase/trypsin (2.4U/0.05%) solution (D4693-1G, Sigma^®^ and 25300-062, Gibco^®^) added to the flask. The flask was incubated at 37 °C, 5% CO_2_ for 1.5 h with gentle agitation (vortex) to achieve a single-cell suspension of epithelial cells.

Trypsin was neutralized with a 1:1 proportion of complete medium. The solution was then centrifuged at 1600× *g* rpm for 5 min, and the supernatant was removed. Medium was added to the remaining pellet (10 mL), and the solution was filtered using a sterile cell strainer to eliminate any undigested tissue/debris. The mitomycin medium was removed from the feeding layer at different time points (Method A vs. B). Isolated cells (5 mL of the medium containing cells) were seeded into a 25 cm^2^ T-flask with the previously cultured feeding layer and incubated at 37 °C with 5% CO_2_ and monitored over time [16].

### 2.5. Passage, Purification, and Cell Line Expansion

The 10 µg/mL mitomycin C solution was applied to the pre-cultivated L929 cells. Subsequently, an EDTA/PBS solution (0.2 g NaEDTA/Lit PBS) was meticulously prepared and filtered. This solution was introduced to the cells (rHFSCs and L929) to facilitate L929 detachment, involving a 5–10 min of incubation at 37 °C, accompanied by mechanical force application, as needed. Following this, 0.05% trypsin-EDTA solution was employed to detach the remaining cells, with the process conducted for 5 min at 37 °C.

The flask underwent a thorough rinsing with culture medium, followed by cell centrifugation at 1600× *g* rpm for 10 min. The supernatant was discarded, and the cells were resuspended in a total volume of 1 mL of medium plus cells. The quantification and determination of cells viability was achieved using the cell countess and a trypan blue exclusion test, aiding in determining the appropriate number for subsequent seeding and cryopreservation. The cells were seeded at a density of 4000 cells/cm^2^ to initiate the desired culture conditions.

### 2.6. Cryopreservation and Thawing

Cells isolated at various passages (P0-P9), underwent cryopreservation and thaw cycles. In culture, the cells underwent enzymatic detachment, utilizing a 0.05% trypsin-EDTA solution (25300062, Gibco^®^), followed by collection and automatic counting. The cells were cryopreserved with complete culture medium without EGF and 10% dimethyl sulfoxide (DMSO—5895690100, SigmaAldrich^®^) in cryovials with at least 1.5 × 10^5^ cells. Subsequently, the cryovials underwent a gradual freezing process (−1 °C/min) within a freezing container containing isopropyl alcohol (Nalgene^®^ Mr. Frosty^®^, Thermo Scientific^®^, Waltham, MA, USA) at −80 °C. Within a maximum period of 3 days, the cryovials were then transferred to canisters within a liquid nitrogen container (−196 °C) for long-term cryopreservation (LS750 Cryogenic Sample Storage, Taylor Wharton^®^, Baytown, TX, USA).

For thawing, the cryovial content was exposed to a water bath (37 °C) for rapid thawing. Within the laminar flow hood, the thawed contents were rapidly collected and centrifuged, and the supernatant discarded. The cells were subsequently resuspended in culture medium, counted, cultured, and maintained under standard conditions.

### 2.7. Cell Behavior in Culture

#### 2.7.1. Growth Curve and Cell Viability

Cells were maintained in culture over 8 passages to establish their growth curve and viability over time. Upon reaching a confluence of 70–80%, a new cell passage was performed. Each passage involved the removal of the culture medium, EDTA/PBS washing for L929 detachment, PBS washing, cell detachment using a 0.05% trypsin-EDTA solution, and a 5 min incubation under standard conditions. The subsequent steps included centrifugation (1600× *g* rpm, 10 min) and the removal of the supernatant. To assess cell number and viability at each passage, the trypan blue exclusion cell assay was employed, and cell counting was conducted using an automated counter (Countess II FL Automated Cell Counter, Thermo Fisher Scientific^®^). The cell areas were measured using ImageJ^®^ 1.54d software version and quantified from 10× microscope images to calculate the mean and SEM values, enabling a comparison of heterogeneity over time.

#### 2.7.2. Population Doubling Time (PDT)

The cells were seeded in 10 wells of a 12-well plate (12 flat test plate, Orange Scientific^®^, Braine-l'Alleud, Belgium) with a density of 5 × 10^4^ cells/cm^2^, and culture medium was added. Over a period of 10 days, the cell count was assessed daily in one well per day. After the 10-day period, the population doubling time (PDT) was determined using the method proposed by Lotfy et al. [29]. The PDT was calculated using the formula PDT = CT/PDN, where CT represents the culture time (in this instance, 10 days), and PDN stands for the population doubling number. The PDN was computed with the formula PDN = 3.32 (log Xf − log Xi), where Xf denotes the cell count at the end of the subculture, Xi is the initial cell number used as the inoculum, and the doubling level of the inoculum used to initiate the subculture was quantified. In this context, since the value of the doubling level was unknown, it was considered as 0. PDT measurements were conducted during Passages P8–P9. Triplicates were considered for each passage.

#### 2.7.3. Colony Forming Unit (CFU) Assay

The CFU assay was conducted by culturing P3 the rHFSCs, until they reached a 70–80% confluence. Subsequently, L929 detachment was performed with EDTA/PBS, followed by enzymatic detachment using a 0.05% trypsin-EDTA solution. The collected cells were subjected to a 1:2 serial dilution in complete medium, allowing for cell plating at densities ranging from 200, 300, 400, to 500 cells per well in a six-well plate. The cells were incubated for 14 days under standard conditions, with daily monitoring to confirm colony development.

After the incubation period, the culture medium was aspirated, and the cells were incubated in 4% formaldehyde for 20 min and then stained with a 0.5% (*v*/*v*) crystal violet solution for 10 min at room temperature. Colony quantification was performed using a magnifying glass (Leica Zoom 2000, Meyer instruments^®^, Houston, TX, USA). Only visible colonies exceeding 1 mm in diameter or comprising more than 50 cells were considered, while overlapping colonies were disregarded. The clonal efficiency, expressed as the percentage of clonogenicity, was calculated using the formula (mean number of counted colonies/total number of seeded cells) × 100. To determine the CFU value, colonies from six wells (*n* = 6) in a six-well plate were counted. This assay was performed according to Penfornis et al. [30].

#### 2.7.4. Differentiation Protocols

##### Adipogenic Differentiation and Oil Red O Staining

Regarding the adipogenic differentiation protocol, 1 × 10^4^ cells/cm^2^ (P3) were seeded in a 12-well plate, and complete culture medium was added. The plate was then incubated under standard conditions for 4 days. After this period, the culture medium in 6 wells was replaced with the complete adipogenesis differentiation medium (StemPro^®^ Adipogenesis Differentiation Kit, Gibco^®^), while 2 wells were designated as controls and maintained with the regular culture medium. Following the manufacturer’s instructions, the media were refreshed every 3–4 days, and the cells were sustained in differentiation for 14 days.

After 14 days, the oil red O staining protocol was executed. The culture and differentiation media were aspirated, and the wells were gently washed with PBS. Cells were fixed with 4% formaldehyde (3.7–4% buffered to pH7, 252,931.1315, Panreac Appli-Chem^®^, Darmstadt, Germany) for 20 min at room temperature, followed by three additional washes with PBS. Oil red O solution (010303, DiaPath^®^, Martinengo, Italy) was added to each well, and the plate was incubated for 20 min at room temperature. Subsequently, oil red O was discarded, and any excess dye was removed through several washes with PBS. PBS was added to each well for visualization. The wells were observed under an inverted microscope. The purpose of this assay was to identify intracytoplasmic lipid vacuoles, characterized by their red coloration upon exposure to the oil red O solution.

##### Chondrogenic Differentiation and Alcian Blue Staining

rHFSCs were seeded in a 96-well plate with a micro-mass culture setup at a density of 2.4 × 10^4^ cells per well (P3), positioned at the center of the well. The plate was kept under standard conditions for 2 h. Following this period, the differentiation medium (StemPro^®^ Chondrogenesis Differentiation Kit, Gibco^®^) was introduced into 8 wells. The medium was refreshed every 2–3 days, and the cells were maintained in these conditions for a span of 14 days. Subsequently, the alcian blue staining protocol was applied. After washing the cells with PBS, they were fixed with 4% formaldehyde for 20 min at room temperature. The wells underwent three PBS washes, and alcian blue solution (119851-27-3, Sigma-Aldrich^®^) was added for 30 min at room temperature. After removing the stain, distilled water was added three times, remaining in the wells to facilitate microscopic visualization. The aim of this assay was to confirm chondrogenic differentiation through the identification of glycosaminoglycans in the extracellular matrix, which will be stained blue with the alcian blue solution.

##### Osteogenic Differentiation and Alizarin Red S/Von Kossa Staining

rHFSCs were initially seeded in a 12-well plate at a density of 8 × 10^3^ cells per cm^2^ (P3), accompanied by culture medium, and incubated for 4 days. Subsequently, the culture medium was replaced with osteogenic medium (StemPro^®^ Osteogenic Differentiation Kit, Gibco^®^), with medium refresh every 3–4 days. The cells were maintained under these conditions for 21 days. Following this, both alizarin red S and Von Kossa staining protocols were implemented. For alizarin red S, the cells underwent washing with PBS, fixation with 4% formaldehyde for 30 min at room temperature, and subsequent washing with distilled water. Alizarin red S solution (2003999, Merck^®^) was added for a 30-minute incubation period. After stain removal, distilled water was added multiple times to eliminate excess, and the wells were observed under the microscope with distilled water. For Von Kossa staining, cells were fixed, dehydrated using increasing concentrations of ethanol, and air-dried. Subsequently, cells were incubated with 2% silver nitrate solution (85193, Sigma Aldrich^®^) for 30 min at room temperature. Following this, cells were treated with sodium thiosulfate 5% (72049, Sigma Aldrich^®^), rinsed, and then microscopically analyzed. The aim of this assay was to determine the capacity for osteogenic differentiation through the identification of calcium phosphate deposits stained with Von Kossa (black staining) and calcium deposits in the extracellular matrix stained with alizarin red (red staining).

### 2.8. Reverse Transcriptase Polymerase Chain Reaction (RT-PCR)

For PCR analysis, rHFSCs P3 were employed. Fifteen target genes, along with the two reference genes—beta-actin (ACTB) and glyceraldehyde 3-phosphate dehydrogenase (GAPDH)—were amplified in separate reaction tubes. The total RNA extraction was conducted using the TRIzol RNA extraction kit, following the manufacturer’s instructions, and cDNA was synthesized using reverse transcriptase. The PCR reaction system included 29 SYBR green mix (10 µL), primer mix (1 µL), template (1 µL), and H_2_O (8 µL). This reaction mixture was loaded into Axygen PCR tubes, briefly centrifuged, and then placed into the real-time PCR instrument, employing the SYBR green method. The thermocycling program consisted of 40 cycles at 95 °C for 15 s, 60 °C for 15 s, and elongation at 72 °C for 20 s. Each cDNA sample underwent processing in triplicate. The copy number in each cDNA sample was determined based on the calibration curve generated by the gene PCR products.

The expression of 15 specific genes was analyzed to identify markers associated with key cellular differentiations, including osteogenic (RUNX2, IBSP), chondrogenic (COL2A1, ACAN), and adipogenic (ADIPOQ, AAK1) differentiations. Additionally, three genes were examined as epithelial stem cell markers (ITGβ1, KRT19, and p63), one gene as a bulge stem cell marker (CD34), and others as markers for the spinous layer (KRT10) and the epithelial basal layer (ITGα6). KRT15 was used as a keratinocyte marker, and two housekeeping genes, GAPDH and ACTB, were used for normalization.

For the gene expression analysis, a Prime PCR Custom Plate 96 Well from Bio Rad Laboratories^®^ was utilized, featuring 15 predesigned primers for the specified genes.

This experimental design enabled a comprehensive exploration of gene expression patterns associated with various cellular processes, providing valuable insights into the differentiation and characterization of cells. The use of housekeeping genes ensured accurate normalization, enhancing the reliability of the obtained gene expression data.

#### 2.8.1. RNA Isolation and cDNA Synthesis

RNA isolation was carried out using the Aurum™ Total RNA Mini Kit (Bio Rad Laboratories^®^, Hercules, CA, USA), following the provided manufacturer’s guidelines. In summary, a pellet containing 2 × 10^6^ cells was lysed with a specific lysis solution, DNA was eliminated using DNAase I enzyme, and the resulting RNA was eluted with 80 μL of an elution solution. The isolated RNA was stored at −80 °C for subsequent use.

Before cDNA synthesis, the RNA’s quantity and purity were assessed through UV-spectrophotometry by measuring A260/A280 (indicating protein contamination) and A260/A230 (indicating contamination with polysaccharides, phenol, and/or chaotropic salts) absorbance on a nanodrop spectrophotometer (Implen GmbH, Isaza^®^, Munich, Germany). Acceptable purity values ranged from 2 to 2.2 for A260/A280 and from 1.8 to 2.2 for A260/A230.

First-strand cDNAs were synthesized using 3.51 μL of total RNA in a final volume of 20 μL, employing the iScript™ cDNA Synthesis Kit (Bio Rad Laboratories^®^), following the manufacturer’s instructions. The entire reaction mix underwent incubation in a thermal cycler (T100™ Thermal Cycler, Bio Rad Laboratories^®^), following the time and temperature guidelines specified in the kit’s instructions.

#### 2.8.2. Quantitative RT-PCR Assay

The RT-PCR assay was conducted using the CFX Connect Real-Time PCR Detection System from Bio Rad Laboratories^®^. Standard PCR conditions were employed with iTaq™ Universal SYBR Green Supermix (Bio Rad Laboratories^®^), following the manufacturer’s recommendations. The real-time PCR detection system was used to read plates containing a mix targeting 15 genes for expression analysis in rHFSCs. Each primer pair aimed at specific genes, and the temperature cycles recommended by the manufacturers were applied.

Upon the completion of the RT-PCR, gene expression analysis was performed. To confirm product specificity, a melting curve analysis was conducted. Threshold cycle (Ct) values of 39 were considered indicative of weak reactions, suggesting minimal target nucleic acid presence or environmental contamination. For each passage, the ΔCt value was determined using the formula ΔCt = Ct(target gene) − Ct(housekeeping gene).

### 2.9. Immunohistochemical Analysis

The rHFSCs at P3 were subjected to immunohistochemical analysis to specific immunomarkers (Table 1). The cells were cultured in 6-well plates (3.84 × 10^4^ cells/well) until a confluence of 70–80% was reached, and immunohistochemical technique was performed using the Novolink™ Polymer Detection Systems kit (Leica Biosystems^®^, Nussloch, Germany), following the manufacturer’s instructions. Details regarding the primary antibodies and antigen retrieval methods are summarized in Table 1. The chosen antibodies aimed to rule out as mesenchymal (vimentin), as well as endothelial (CD31) cells’ histogenesis. Additionally, the non-expression of neural markers (synaptophysin) and smooth muscle markers (α-muscle actin) was confirmed, to rule out the putative isolation of cells from the nerve plexuses involving the hair follicle or the piloerector muscle, respectively. A specific surface marker for rHFSCs was also considered (p63) [31,32]. To confirm the cells stemness, the c-kit immunomarker was also employed. The observation, evaluation, and photography of the samples were performed using the Nikon^®^ Eclipse E600 microscope and Imaging Software NIS-Elements F Ver4.30.01 (Laboratory Imaging^®^, Prague, Czech Republic). Immunopositivity was considered when distinct nuclear or cytoplasmic immunoreactivity was evident in at least 5% of the cells (0, negative; +).

### 2.10. rHFSCs Secretome Production and Analysis

To identify specific chemokines and growth factors produced and released by rHFSCs, the secretome was subjected to analysis. The conditioning process involved using cells in early passages (P3 and P5) to assess if there were any statistical differences between these passages that could influence the selection of a passage for subsequent in vivo assays. Upon reaching 70–80% confluence, the culture medium was removed, and the culture flask was gently washed with PBS 3 times. Two additional washes with DMEM culture medium followed, and then basal culture medium without antibiotic, antimycotic, or FBS supplementation was added to the flask (DMEM-F12 11039-021 Gibco^®^). The culture was incubated under standard conditions for 48 h. After this incubation period, the secretome, enriched with factors secreted by the cells, was collected and centrifuged, and the supernatant was retained. The secretome was stored at −20 °C and, subsequently, subjected to analysis using multiplexing LASER bead analysis (Eve Technologies, Calgary, AB, Canada) to explore a specific panel of biomarkers (Rat Cytokine/Chemokine 27-Plex Discovery Assay^®^ Array (RD27) and transforming growth factor beta (TGFβ 3-Plex Discovery Assay^®^ Multi Species Array (TGFβ1-3)). The studied biomarkers included EGF, vascular endothelial growth factor (VEGF), interleukin (IL)-6, IL-1α, IL-1β, IL-2, IL-4, IL-5, IL-10, IL-12p70, IL-13, IL-17A, IL-18, regulated upon activation, normal T cell expressed, and presumably secreted (RANTES), monocyte chemoattractant protein-1 (MCP-1), tumor necrosis factor-alpha (TNFα), granulocyte colony-stimulating factor (G-CSF), eotaxin, fractalkine, leptin, interferon-gamma (IFNϒ), interferon-gamma inducible protein (IP-10), human growth regulated oncogene/keratinocyte chemoattractant/cytokine-induced neutrophil chemoattractant-1 (GRO/KC/CINC-1), granulocyte macrophage colony-stimulating factor (GM-CSF), LIX, macrophage inflammatory protein (MIP) 1α, MIP-2, TGFβ1, TGFβ2, and TGFβ3. Three independent samples were analyzed for each passage.

### 2.11. Statistical Analysis

The statistical analysis was conducted using GraphPad Prism version 8.00 for Windows (GraphPad Software, La Jolla, CA, USA). Data, when appropriate, are presented as mean ± standard error of the mean (SEM). Group comparisons were executed through parametric tests. A significance threshold of <0.05 was considered statistically significant. The significance of the results is indicated by symbols (*), with (*) corresponding to 0.01 ≤ *p* < 0.05, (**) to 0.001 ≤ *p* < 0.01, (***) to 0.0001 ≤ *p* < 0.001, and (****) to *p* < 0.0001.

## 3. Results

### 3.1. Mitomycin C Concentration Determination Assay with L929 Cells

**Figure 3:**
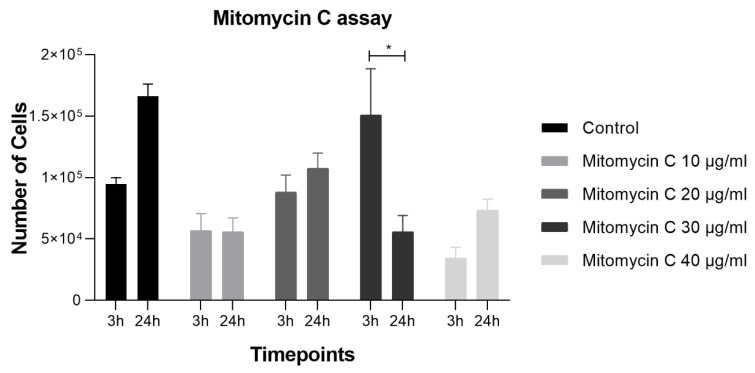
Number of cells at 3 h and 24 h post-treatment with mitomycin C at 10 μg/mL, 20 μg/mL, 30 μg/mL, and 40 μg/mL. The significance of the results is indicated by symbols (*), with (*) corresponding to 0.01 ≤ *p* < 0.05.

Figure 3 demonstrates the visual representation of the number of cells counted at 3 h and 24 h post mitomycin C treatment. The cell number increased from 3 h to 24 h in the control group, as expected. The group treated with 10 μg/mL mitomycin C showed no significant difference in the cell number between 3 h and 24 h post-treatment. The group treated with 20 μg/mL and 40 μg/mL mitomycin C demonstrated a slight increase in cell number at 24 h compared to 3 h. In contrast, the group treated with 30 μg/mL mitomycin C exhibited a significant decrease in the cell number compared to the control at 3 h, with statistical significance (*p* < 0.05).

**Figure 4:**
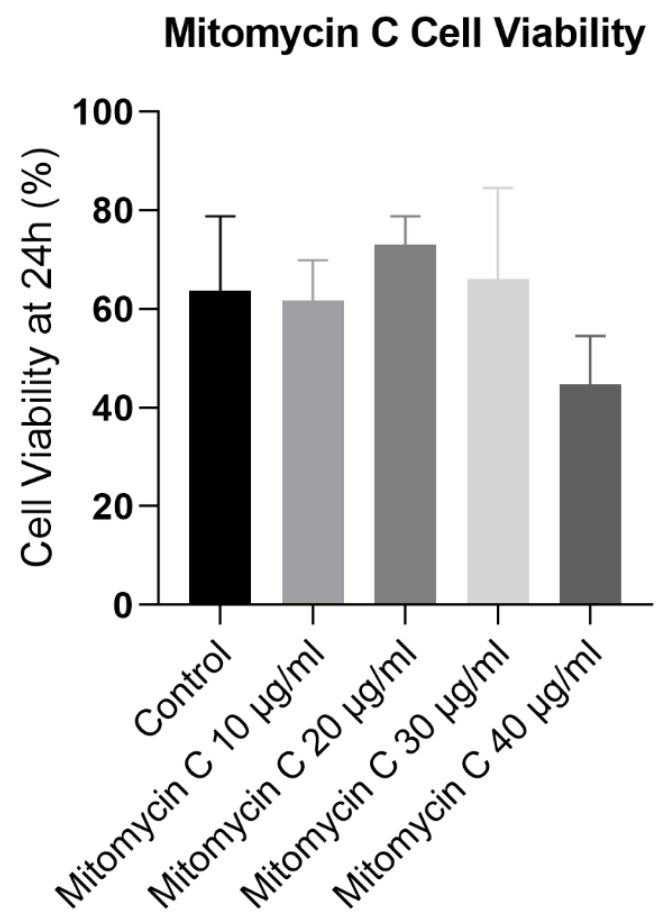
L929 viability at 24 h post mitomycin C treatment.

In Figure 4, it can be confirmed that cell viability showed no significant differences between the control and mitomycin C-treated groups at concentrations of 10, 20, and 30 μg/mL. However, the group treated with 40 μg/mL mitomycin C exhibited a slight decrease in cell viability compared to the control. These results demonstrate that mitomycin C treatment had concentration-dependent effects on L929 cell proliferation. Concentrations of 20 and 40 μg/mL promoted cell proliferation, while 30 μg/mL had an inhibitory effect, diminishing the cell number over time. These findings suggest that mitomycin C can effectively halt cell division in L929 cells at a concentration of 10 μg/mL without compromising cell viability, as indicated by the lack of significant differences in viability at lower concentrations. The observed increase in cell number at 24 h in the control group likely reflects normal cell growth and proliferation. The sustained increase in the cell number in the presence of 20 and 40 μg/mL mitomycin C suggests that these concentrations do not effectively block cell division. However, the significant decrease in cell number at 24 h in the presence of 30 μg/mL mitomycin C indicates a potent inhibitory effect on cell proliferation, potentially due to some cytotoxicity. Importantly, the 10 μg/mL concentration resulted in a cell division arrest, without a significant reduction in cell viability. In conclusion, the concentration of 10 μg/mL effectively halted cell division, making it the selected concentration to use in the feeding layer for the rHFSCs.

### 3.2. Isolation and Expansion of rHFSCs

**Figure 5:**
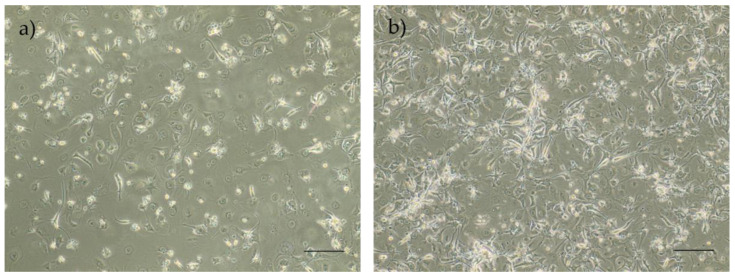
Representative images of P0 rHFSCs with P6 L929 feeding layer, previously treated with mitomycin C solution isolated with Method A (**a**) and Method B (**b**). Magnification: 40×; scale bar: 100 µm.

The methods used to isolate the cells both showed efficacy and allowed observation of the cells growing in the feeding layer (Figure 5). However, Method B resulted in a higher yield of isolated cells, with better performance over time. Consequently, cells isolated using this method were selected for subsequent assays.

### 3.3. Growth Kinetics and Cell Viability

**Figure 6:**
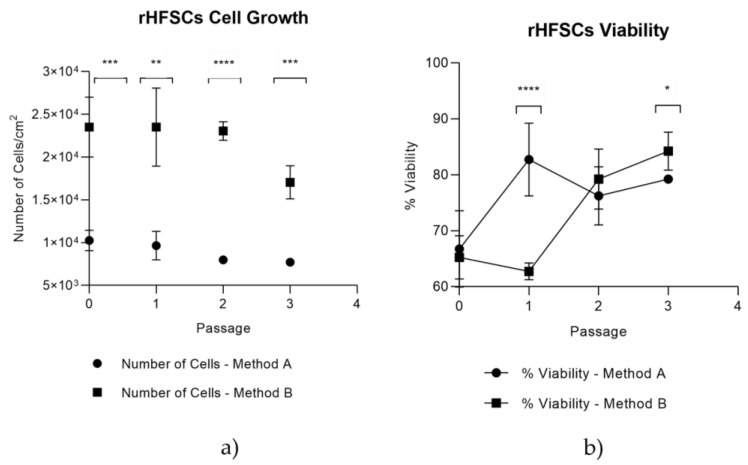
rHFSCs comparison between isolation Methods A and B on the 4 initial passages—(**a**) rHFSC cell growth comparison and (**b**) rHFSC viability comparison. The significance of the results is indicated by symbols (*), with (*) corresponding to 0.01 ≤ *p* < 0.05, (**) to 0.001 ≤ *p* < 0.01, (***) to 0.0001 ≤ *p* < 0.001, and (****) to *p* < 0.0001.

Isolation Methods A and B were compared between P0 and P4 regarding cell growth and viability, with Method B selected as the superior approach, as it demonstrated a higher cell proliferation and increased viability from Passage 2, with statistical significance (Figure 6a,b).

**Figure 7:**
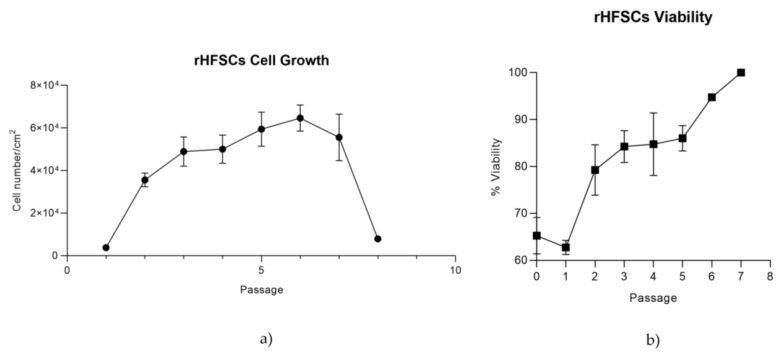
rHFSC growth curve over 8 passages (**a**) and rHFSCs viability over 8 passages (**b**).

The two graphics shown above illustrate the growth curve and viability of rHFSCs over eight passages. In Figure 7a, cell proliferation increases significantly in the early passages, reaching its peak at Passage 6, after which the growth rate declines. In contrast, Figure 7b shows a continuous increase in cell viability as the passages progress, reaching 100% by Passage 8.

### 3.4. Cryopreservation and Thawing

**Figure 8:**
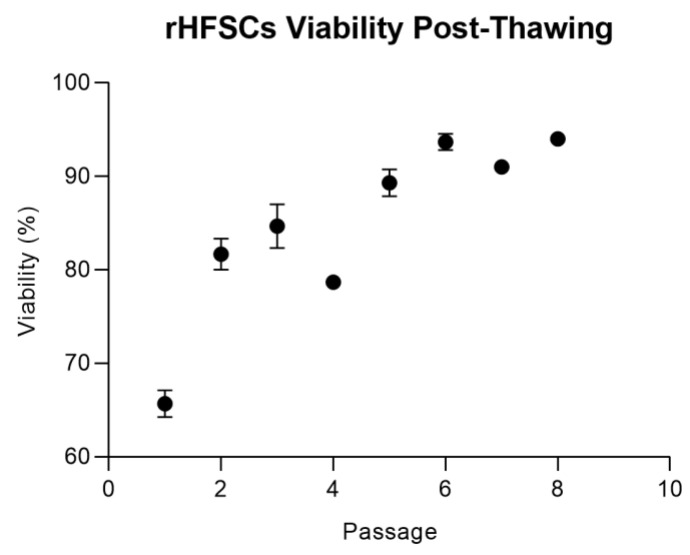
rHFSC viability over time, post-thawing (mean ± SEM).

Cryopreserved and thawed cells exhibited consistent performance without discernible changes. Regardless of the passage at which cryopreservation occurred, the cells adhered to the plastic surface within a few hours post-thawing, and their viability consistently exceeded 66%, showing an improvement with successive passages (Figure 8). Confluence, reaching 70–80%, occurred between 3 and 4 days, and the expected morphology was observed.

### 3.5. Cell Behavior in Culture

**Figure 9:**
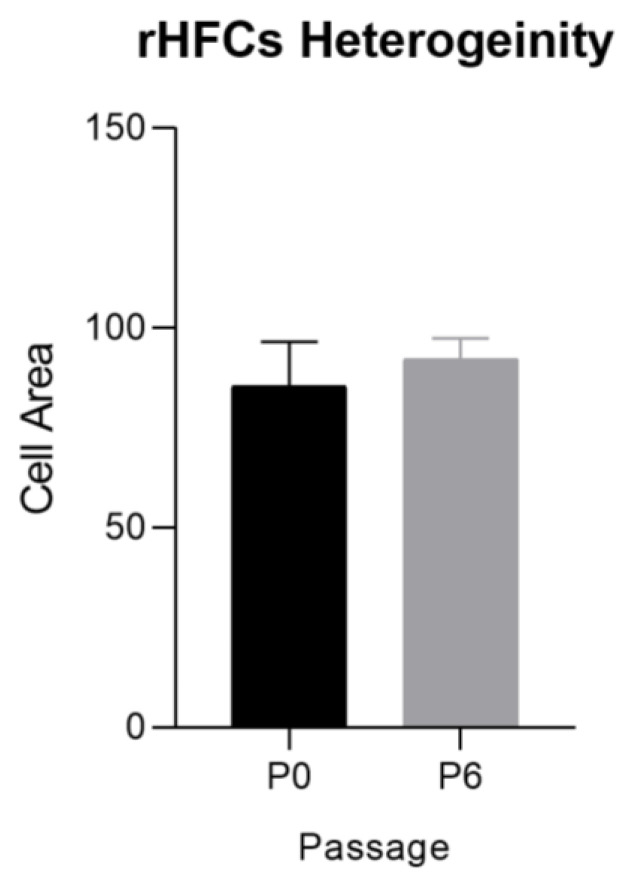
rHFSC heterogeneity over time (mean ± SEM).

At the beginning of the culture, the cells exhibited a heterogeneous morphology, as the culture was not yet purified. Following the initial passages, the cultures transitioned toward a more homogeneous morphology. This shift is evident in Figure 9, where the cells’ mean area in P0 displays a higher standard error of the mean (SEM) compared to P6. Culture cells exhibited clear plastic adhesion. The observed variations among different experiments likely stemmed from differences in the efficiency of hair follicle dissociation. This transition in culture characteristics suggests a convergence towards a more uniform cell population after the first passages. In earlier passages, the adhesion time was extended, and cell growth and proliferation were comparatively slower.

#### 3.5.1. PDT

**Figure 10:**
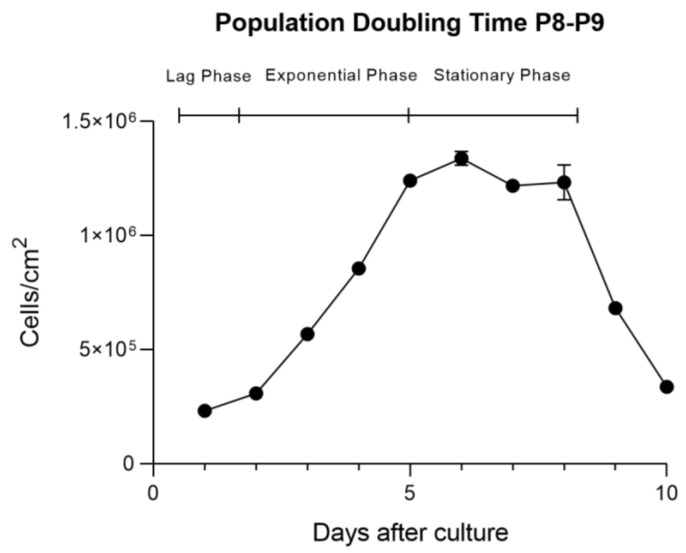
rHFSC cell growth over 10 days of culture in P8–P9 (mean ± SEM) with the lag, exponential, and stationary phases.

**Figure 11:**
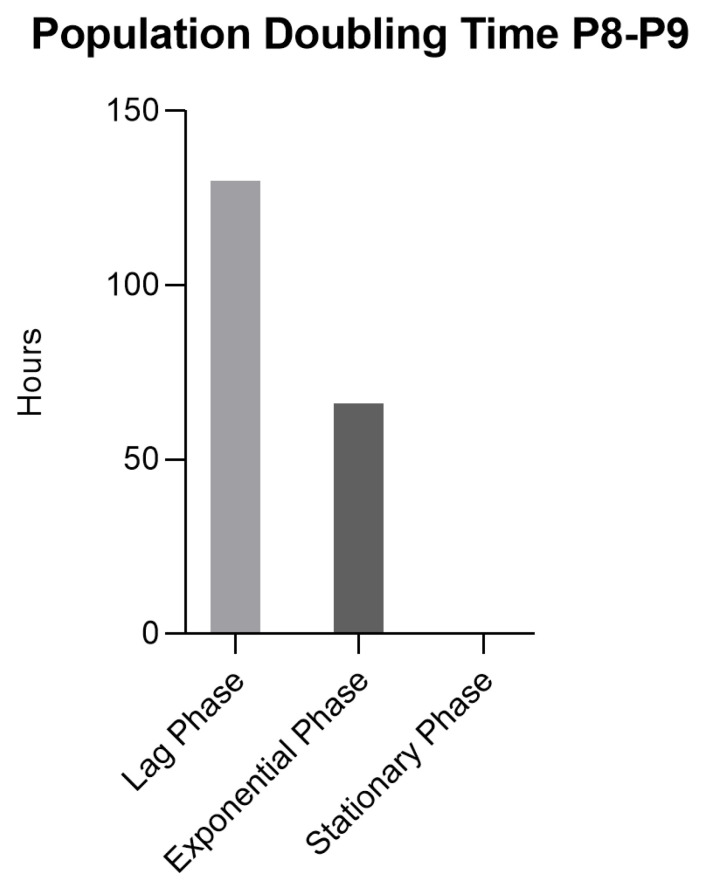
rHFSC PDT for each phase (lag, exponential, and stationary).

The PDT of rHFSCs was determined for P8–P9, and the three phases traditionally attributed to cells in culture were easily identified, as the lag phase occurred between P1 and P2, the exponential phase between P2 and P5, and the stationary phase between P5 and P8 (Figure 10). The mean value of PDT was 68.38 ± 19.12 h, and for each phase, it is represented in Figure 11. The lag phase took approximately 130 h to double the population, while the exponential phase took around 66 h. The stationary phase, as expected, had a negligible doubling time.

#### 3.5.2. Colony Formation Assay (CFU)

**Figure 12:**
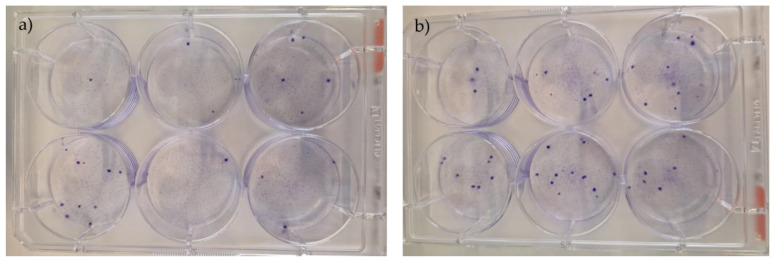
Results of the rHFSC CFU assays (P3), with initial seeding densities of 200/300 (**a**) and 400/500 (**b**).

**Figure 13:**
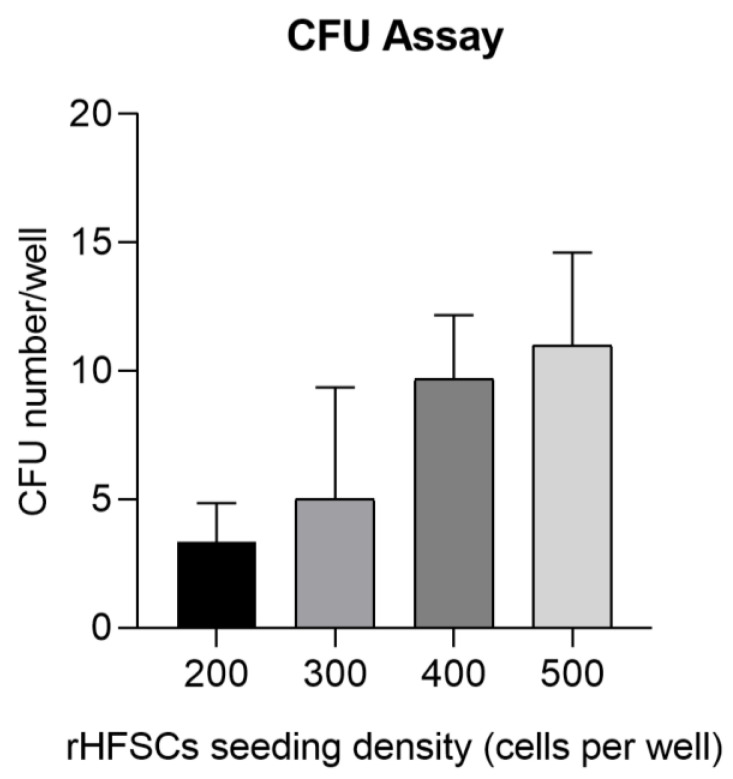
Results of the rHFSC CFU with initial seeding densities of 200, 300, 400, and 500 cells per well.

The CFU assays provided confirmation of the rHFSC capacity to generate new colonies from individual cells. Following a 14-day incubation period, an average of 8.63 ± 2.86 colonies exhibiting spindle morphology were identified per well. The clonogenicity percentage for 200 seeded cells was 3.33 ± 1.53; 300 seeded cells was 4.67 ± 4.73; 400 seeded cells was 11.0 ± 2.65; and 500 seeded cells was 9.67 ± 2.52 (Figure 12 and Figure 13).

#### 3.5.3. Differentiation Protocols

##### Adipogenic Differentiation and Oil Red O Staining

**Figure 14:**
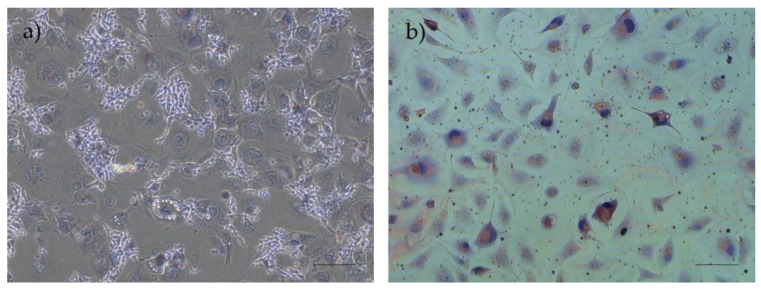
Results of adipogenic differentiation assays in rHFSCs P3: (**a**) control and (**b**) with red-stained lipid vacuoles in the cytoplasm. Magnification: 40×; scale bar: 100 µm.

The confirmation of the cells’ adipogenic differentiation ability was established through the observation of morphological changes, characterized by the presence of large cells with a rounded shape, and the detection of red-stained lipid vacuoles in the cytoplasm, resulting from exposure to the oil red O solution (Figure 14).

##### Chondrogenic Differentiation and Alcian Blue Staining

**Figure 15:**
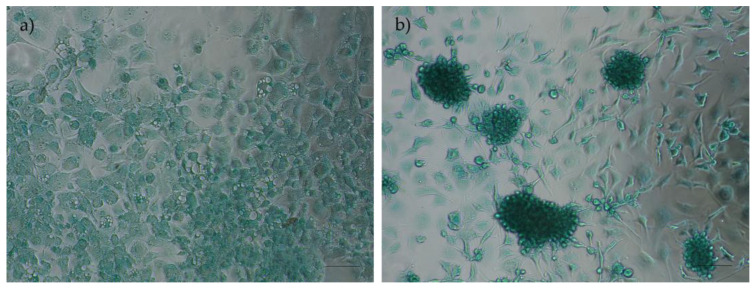
Results of chondrogenic differentiation assays in rHFSCs P3: (**a**) control and (**b**) chondrogenic aggregates and extracellular matrix staining in blue. Magnification: 40×; scale bar: 100 µm.

The chondrogenic differentiation of rHFSCs was verified through the observation of chondrogenic aggregates and extracellular matrix staining in blue, attributed to the exposure of proteoglycans to the alcian blue solution (Figure 15).

##### Osteogenic Differentiation and Alizarin Red S/Von Kossa Staining Assays

**Figure 16:**
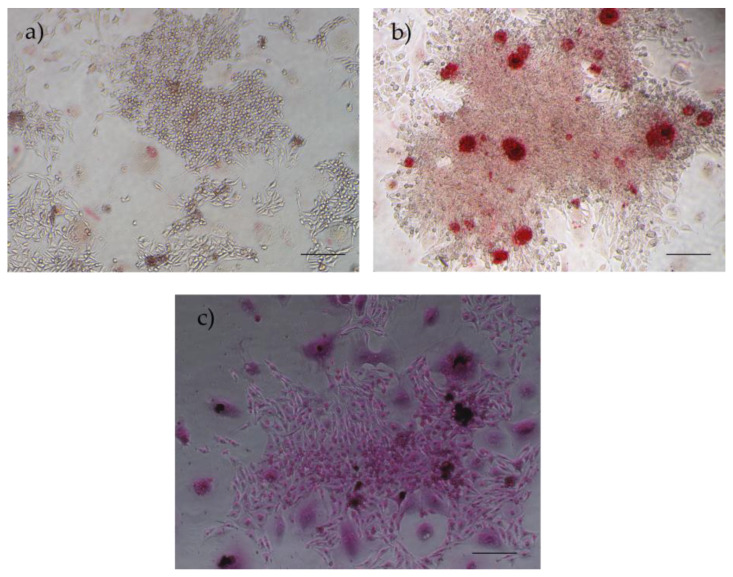
Results of osteogenic differentiation assays in rHFSCs P3: (**a**) control; (**b**) alizarin red staining—presence of calcium deposits; (**c**) Von Kossa staining—presence of dark deposits (calcium and phosphate). Magnification: 40×; scale bar: 100 µm.

The confirmation of cells’ osteogenic differentiation capacity was achieved through the identification of cells containing calcium, suggestive of osteocytes, and the detection of extracellular calcium deposits stained red, attributable to exposure to the alizarin red solution. Also, the Von Kossa staining demonstrated the presence of dark deposits. These deposits represent calcium and phosphate, indicating the presence of mineralized matrix produced during osteogenic differentiation (Figure 16). Alizarin red detects calcium deposits, while Von Kossa identifies both calcium and phosphate, offering a broader understanding of mineralization. Co-staining validates that the mineralization observed with alizarin red is associated with actual bone formation. Additionally, Von Kossa reveals the spatial distribution of the mineralized matrix, enhancing the analysis of bone architecture [36].

### 3.6. RT-PCR

**Figure 17:**
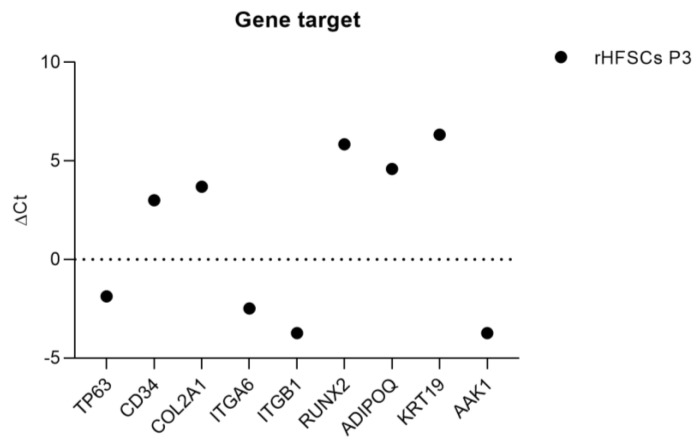
ΔCt values for each gene under study. Higher ΔCt values demonstrate lower expression (mean ± SEM).

Table 2 presents the average Ct values and ΔCt values for the genes analyzed via PCR for rHFSCs at P3. The purity of the samples was confirmed, allowing for their use in subsequent phases of the study. *ACTB* was used as the reference gene, showing consistent expression. The genes were divided into highly expressed genes (Ct < 29), moderately expressed genes (29 ≤ Ct ≤ 35), and lowly expressed genes (Ct ≥ 35). Regarding the ΔCt values, a negative ΔCt indicates that the target gene is expressed at a higher level than the reference gene. This can suggest that the target gene is upregulated, which may be relevant in various biological contexts, such as developmental processes, responses to stress, or disease states. *p63* and *KRT19* were expressed (moderately and lowly), confirming the identity of the cells as epithelial stem cells. *CD34*, a well-known marker for bulge stem cells, was also expressed, further supporting this identification. The integrins *ITGα6* and *ITGβ1*, which are crucial for cell adhesion and signaling, demonstrated moderate expression consistent with the basal characteristics of these cells. Additionally, *COL2A1*, a marker associated with chondrogenesis, was expressed, suggesting a potential differentiation toward cartilage. However, the lack of expression of *ACAN*, another chondrogenic marker, indicates that this pathway may not be fully activated. Notably, *RUNX2* displayed expression and a potential commitment to osteogenic differentiation, while *IBSP*, another osteogenic marker, was not expressed. Moreover, the presence of *ADIPOQ/AAK1* suggests the possibility of adipogenic differentiation. Overall, these results suggest that the rHFSCs (P3) show characteristics of multipotent stem cells, with potential for differentiation toward multiple lineages, including chondrogenic, osteogenic, and adipogenic pathways. Additionally, the lack of *KRT14, KRT10*, and minimal *KRT15* expression supports the conclusion that these cells have not retained full bulge-specific characteristics (Figure 17).

### 3.7. Immunohistochemical Analysis

**Figure 18:**
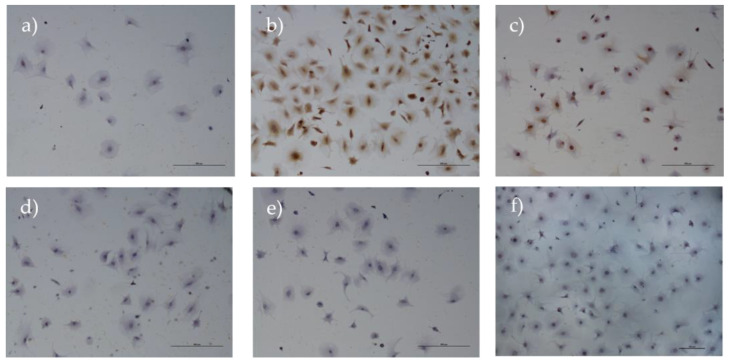
Results of the immunohistochemical analysis of isolated rHFSCs at P3. The cells are negative for CD31 (**a**), synaptophysin (**d**), vimentin (**e**), and alpha-smooth muscle actin (**f**), but positive for both cKit (**b**) and p63 (**c**).

The selected antibodies were carefully chosen to exclude the possibility of mesenchymal origin, as indicated by the negative expression of vimentin. Furthermore, the endothelial origin was dismissed by assessing the absence of CD31 expression, and the non-expression of neural markers, such as synaptophysin, confirmed the cells’ nonneural lineage. The non-expression of α-muscle actin confirmed the absence of smooth muscle-derived cells. Additionally, a specific surface marker for rHFSCs, namely p63, was employed to further validate the cell type under investigation, which showed positive immunolabeling. Positive c-Kit-immunoexpression is indicative of their stemness and potential ability to differentiate into several cell types within the hair follicle structure (Figure 18).

### 3.8. rHFSCs Secretome Analysis

**Figure 19:**
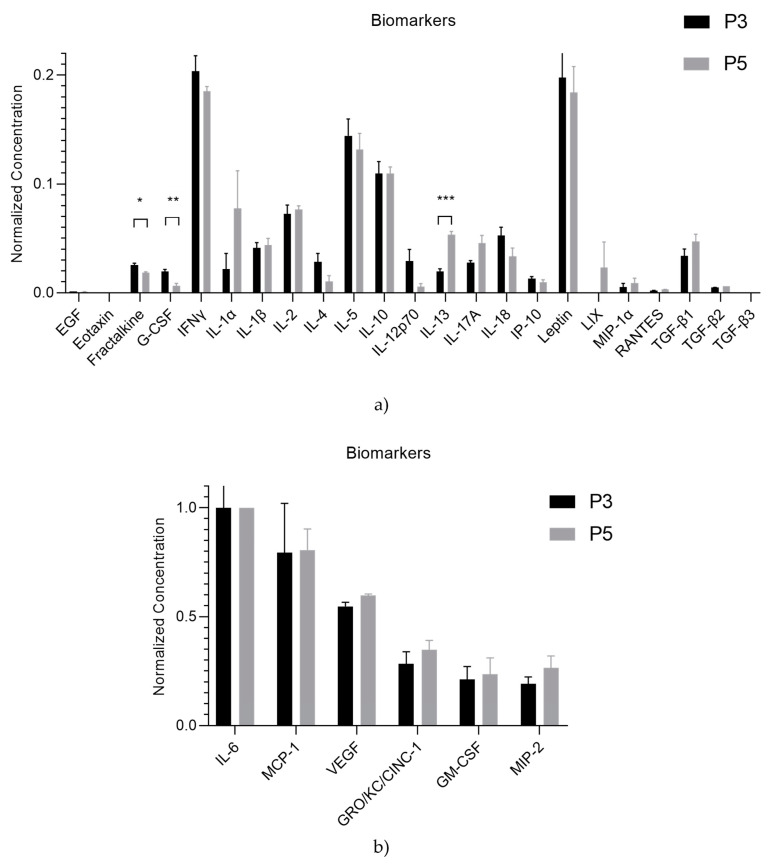
(**a**,**b**) Normalized concentration of each biomolecule present in the rHFSCs secretome (mean ± SEM). The significance of the results is indicated by symbols (*), with (*) corresponding to 0.01 ≤ *p* < 0.05, (**) to 0.001 ≤ *p* < 0.01, and (***) to 0.0001 ≤ *p* < 0.001.

The mean concentration values of each biomarker in the secretome under analysis are presented in Table 3 and Figure 19a,b.

## 4. Discussion

RHFSCs have garnered significant attention due to their easy accessibility and availability, making them a promising candidate for regenerative therapies [16]. This study’s main goal was to establish new methods for their collection, isolation, characterization, and storage, as well as analyzing their secretome regarding their wound healing properties, for the first time.

A simple and easy collection method was established, and the isolation was performed under aseptic conditions, using antibiotics and antimycotics to prevent contamination. The methodology was described as detailed as possible to ensure the future reproducibility and reliability of the results.

The protocol developed by the authors demonstrates several differences when compared to methods developed by other research groups [16,37,38]. The protocol described in the study adopts a comprehensive and meticulous approach to isolating rat hair follicle stem cells (rHFSCs), incorporating key elements for tissue preparation, enzymatic digestion, and subsequent cell culture. Unlike the protocol developed by Sieber-Blum et al., which emphasizes minimal disruption using a tungsten needle, the described method employs dispase and trypsin solutions for enzymatic tissue dissociation, facilitating the creation of a single-cell suspension. In addition, other protocols involve culturing the hair follicle bulges in Matrigel or collagen-coated plates with specialized media for specific cell phenotypes, as the current protocol incorporates a mitomycin-treated L929 cell feeding layer, providing a unique microenvironment tailored for rHFSC isolation. This is further refined by the comparison of two preconditioning methods (A and B) to enhance cell yield and viability. The complete culture medium used was also more complex than the ones described in other protocols, demonstrating the novelty of the work performed.

After isolating and culturing, the cells displayed typical epidermal stem cell features, such as good adhesion ability and a decline in the proliferation capacity with increasing passage numbers [16,39].

Isolation Method B, where the culture medium conditioned pre-isolation, demonstrated superior results compared to Method A (non-conditioning), regarding cell viability and growth kinetics, a finding that aligns with initial expectations, due to the presence of secreted molecules that facilitate cell adhesion, proliferation, and differentiation, thereby creating a more supportive environment for the cells during the isolation process. The analysis of P0–P4 focused on comparing isolation Methods A and B, as early passages closely resemble the original tissue state and are ideal for assessing initial isolation efficacy and selecting the superior method for long-term studies. This approach demonstrated better cell yield and viability over time, and those cells were subsequently used in all following assays.

Cryopreservation is an important aspect for cell preservation, ensuring their availability for therapeutic applications. In this study, DMSO was used as the cryoprotectant due to its ability to maintain high cell viability by preventing intracellular ice formation. Although DMSO has potential tumorigenic and cytotoxic effects above 4 °C, proper thawing and removal protocols minimize these risks [40,41,42]. It is vital that cells retain their culture behavior, immunomodulatory properties, and ability to differentiate after thawing, as verified in this study.

Growth kinetics were determined through both the growth curve and PDT. While the growth curve tracks cell numbers at the time of passage, PDT measures the time required for cells to double within the same passage, offering a more accurate indicator of cell performance. PDT is directly related to genetic stability and senescence, with lower PDT indicating faster cell growth [6,43]. After identifying Method B as superior, growth curves and viability assays were extended to P8 to evaluate long-term cell behavior. P8 was chosen to assess the cumulative effects of prolonged culture, such as senescence or adaptation, ensuring the cells’ robustness and suitability for long-term applications. Growth curves followed typical patterns seen in other species and cells lines, with three phases: lag, exponential growth, and stationary [44]. Viability over time was high even at higher passages; however, from P7 onward, there was a gradual reduction in proliferation, though the cells still proliferated rapidly [45].

CFUs were used to assess self-renewal capacity, and the cells showed good clonogenicity, forming several spindle-shaped colonies, similar to findings in other studies [30]. The CFU assay demonstrated that rHFSCs possess strong colony-forming and proliferative potential, confirming their stem cell-like properties. The results support our hypothesis that the isolation and culture methods used effectively preserve and enhance the proliferative capacity of rHFSCs, which is essential for their potential use in skin regeneration therapies. These findings validate the chosen protocol and highlight the suitability of rHFSCs for future regenerative applications.

In this study, after implementing specific differentiation protocols, rHFSCs demonstrated their capacity for adipogenic, osteogenic, and chondrogenic differentiation in vitro, confirming the multipotent nature of these cells [46,47].

The RT-PCR data, in conjunction with established knowledge about hair follicle stem cell (HFSC) markers, provide several insights into the cell population being studied. p63 and *ITGβ1* presence indicate that the cells are epithelial stem cells, as these genes are specific markers. p63 is a specific hair follicle marker associated with a transcription factor that distinguishes basal stem cells from transient amplifying progeny; *KRT19* is expressed in the bulge and hair germ; and *ITGβ1* is vital for maintaining stem cell adhesion to the basement membrane. *ITGα6* is highly expressed in bulge cells and indicates the epithelial basal layer origin [15,39,48,49,50,51,52]. The non-expression of *ACAN* (chondrodiferentiation marker) and IBSP (osteogenic marker) were unexpected considering the capacity for multipotency described above; however, *RUNX2, COL2A1*, and *ADIPOQ/AAK1*, markers of osteogenic, chondrogenic, and adipogenic differentiation, respectively, suggest a potential tri-differentiation capacity, indicating that these cells possess broader multipotency [53,54,55]. The non-expression of *ACAN*, while *COL2A1* is expressed, can be due to the regulatory role of transcription factors, like SOX9. Under specific conditions, SOX9 can inhibit *ACAN* transcription. Also, *COL2A1* is typically expressed during the early stages of chondrocyte development, while *ACAN* is more prominent in later stages, indicating a temporal regulation influenced by *SOX9*. Furthermore, signaling pathways, such as TGF-β and WNT, interact with SOX9 to refine the expression of these extracellular matrix components [56,57,58]. In addition, the non-expression of *IBSP* can be due to its typical role in the later stages of osteoblast differentiation [59,60,61]. While *RUNX2* is known to induce the expression of *IBSP*, it primarily acts during early osteoblast development. Moreover, signaling pathways, such as WNT signaling, are also important in regulating osteoblast maturation and may contribute to the absence of IBSP at this stage [59,60,61]. *CD34*, a well-established bulge stem cell marker in mouse and rat hair follicles, confirms that the population retains key HFSC characteristics [16,48,50,52,62]. The non-expression of *KRT14* (basal keratinocyte marker) and *KRT15* (keratinocyte marker) suggest that the cells origin is from the folliculum and not from the epidermis [48,49,63,64]. In addition, the non-expression of *KRT10* (spinous layer marker) further supports the conclusion that the rHFSCs have not initiated differentiation into mature keratinocytes or other terminally differentiated cell types [48,64,65].

The immunohistochemical results indicate that the rat hair follicle stem cells express markers associated with epithelial progenitor cells (p63) and stemness capacity (c-Kit) and lack markers for endothelial (CD31), neural (Synaptophysin), mesenchymal (Vimentin), and muscle (α-SMA) lineages. This confirms that they are epithelial stem cells, likely involved in the regeneration of the hair follicle and surrounding skin tissue. These results corroborate the PCR results and confirm that the cells isolated are in fact rat hair follicle stem cells [15,16,49].

The use of secretome offers several advantages over living cells for therapeutic purposes. First, it eliminates the risk of immune rejection, making them safer for allogeneic treatments with no risk of tumor development. They also present fewer regulatory and ethical hurdles, as they avoid the need for live cell cultures or genetic manipulation. Furthermore, they are easier to store and transport and have a longer shelf-life. Additionally, they provide targeted healing without the complexity of maintaining live cells, making them a more efficient and safer option for treatment [66,67,68,69]. Therefore, the rHFSCs’ secretome was analyzed to determine the presence of various biomolecules, such as interleukins, growth factors, chemokines, and immunomodulatory and immunosuppressive factors, which were present in distinct concentrations. Although some of them are expressed in a low concentration, it is not unexpected and does not necessarily diminish their therapeutic efficacy. This is because the effective therapeutic concentrations of these factors in a delivery system typically fall within low yet significant ranges [6].

Regarding the biomolecules identified in the secretome: EGF promotes re-epithelialization and the formation of granulation tissue, while VEGF facilitates angiogenesis and supports granulation tissue formation. IL-10 reduces excessive inflammation, and transforming growth factor beta (TGF-β)1, TGF-β2, and TGF-β3 enhance the formation of granulation tissue. Meanwhile, IL-10 functions as an anti-inflammatory cytokine [70]. IL-6 influences macrophage polarization and contributes to fibrosis through its interaction with TGF-β and IL-17A, which promote collagen deposition and fibroblast differentiation. IL-4 and IL-13 support M2 macrophage polarization, aiding repair, while IL-17A enhances the fibrotic feedback loop with IL-6 and TGF-β [71]. RANTES and MCP-1 recruit immune cells to the wound site. TNF-α drives inflammation, G-CSF boosts neutrophil function, and eotaxin attracts eosinophils. Fractalkine aids in immune cell recruitment, while GM-CSF stimulates macrophage proliferation. GRO/KC/CINC-1 attracts neutrophils, and IFN-γ enhances macrophage activation. IL-1α and IL-1β are crucial pro-inflammatory cytokines, IL-2 supports T cell proliferation, and IL-5 activates eosinophils. IL-12p70 and IL-18 enhance inflammatory responses, and IP-10 attracts immune cells. Leptin modulates immune responses, LIX recruits neutrophils, and MIP-1α and 2 facilitate immune cell recruitment and inflammation. Together, these molecules coordinate the immune response, inflammation, and tissue regeneration necessary for effective wound healing [21,23,65,72,73,74].

The rHFSCs exhibited a dynamic secretome profile, as they aged from P3 to P5. Fractalkine and G-CSF expression between the passages diminished, while IL-13 expression was enhanced in P5, with a statistical difference. This indicates that, over successive cell passages, there is a decrease in pro-inflammatory cytokines (Fractalkine, G-CSF) and an increase in anti-inflammatory IL-13, suggesting a shift in the cells’ immune-modulatory behavior. This may reflect cellular adaptation or 2D conformation that could have implications for how these cells behave in experiments or therapeutic applications, particularly in wound healing, where different stages of healing require different cytokine and growth factor environments.

Additionally, the elevated expression of IL-6, MCP-1, VEGF, GRO/KC/CINC-1, GM-CSF, and MIP-2 suggests a predominant pro-inflammatory and immune-stimulatory environment, characterized by heightened immune cell recruitment, inflammation, and a strong emphasis on tissue repair and angiogenesis, important for wound healing.

## 5. Conclusions

rHFSCs have emerged as a promising therapeutic option in regenerative medicine due to their accessibility, ease of collection, and significant regenerative potential. In this study, a comprehensive protocol was developed for the isolation, culturing, cryopreservation, and thawing of rHFSCs. Through the application of this method, the biological characterization of these cells was performed, confirming the expression of several specific genes and their ability to tri-differentiate. Specific surface markers were also identified, further validating the stemness of rHFSCs and confirming their hair follicle bulge origin, making them suitable candidates for therapeutic applications. These cells’ in vitro behavior was also determined over time.

Cell therapies hold great potential for regenerative medicine; however, challenges remain regarding their safety, process standardization, and methods of delivering cells to injured tissues. These concerns have led to increased interest in the therapeutic potential of paracrine factors secreted by stem cells. In the context of rHFSCs, their secretome was studied, leading to the identification and quantification of various biomarkers linked to wound healing. This discovery opens the possibility of utilizing the rHFSCs’ secretome in future regenerative therapies. Moreover, the potential applications of rHFSCs extend beyond wound healing to other regenerative purposes, making them a versatile tool in tissue repair and regeneration.

This research led to the development of a novel and effective isolation technique for rHFSCs, by systematically comparing two innovative isolation methods and determining the superior approach. The study not only optimized the isolation process but also fully characterized the isolated cells, providing comprehensive insights into their biological properties. Additionally, the wound healing potential of these cells was rigorously evaluated, demonstrating their suitability for future applications in skin regeneration therapies. A pioneering aspect of this work was the detailed analysis of the rHFSCs’ secretome, offering new insights into its composition and potential therapeutic applications. These findings represent a significant advancement in the field of regenerative medicine and underscore the utility of rHFSCs for clinical applications.

## Figures and Tables

**Table 1:** Immunohistochemical analysis—antibodies investigated, dilutions, and antigen retrieval methods.

Antibody	Clonality	Manufacturer	Dilution	Antigen Retrieval
c-Kit	Polyclonal	DAKO^®^, Agilent Technologies^®^, Santa Clara, CA, USA	1/450	20 min Dako Target Retrieval Solution/Water Bath 100 °C [33]
Vimentin	Clone V9	DAKO^®^, Agilent Technologies^®^	1/500	20 min Dako Target Retrieval Solution/Water Bath 100 °C [33]
CD31	Clone JC70A	DAKO^®^, Agilent Technologies^®^	1/50	30 min Pepsine 0.4%/Incubator 37 °C [33]
p63	Clone 4A4	Lab Vision Corporation^®^, Fremont, CA, USA	1/200	20 min Dako Target Retrieval Solution/Water Bath 100 °C [34]
α-Muscle Actin	Clone HHF35	DAKO^®^, Agilent Technologies^®^	1/500	20 min Dako Target Retrieval Solution/Water Bath 100 °C [35]
Synaptophysin	Clone SP11	NeoMarkers^®^, Fremont, CA, USA	1/150	20 min Dako Target Retrieval Solution/Water Bath 100 °C [33]

**Table 2:** Average Ct and ΔCt values for genes under study. nd = nondefined.

Target Gene	Ct Average	ΔCt
*KRT14*	nd	nd
*p63*	31.13 ± 0.09	−1.87
*CD34*	36.01 ± 0.42	3.01
*COL2A1*	36.69 ± 0.13	3.70
*ITG* *α6*	30.52 ± 0.1	−2.48
*ACAN*	nd	nd
*ITG* *β1*	29.27 ± 0.12	−3.73
*RUNX2*	38.85 ± 0.02	5.85
*KRT10*	nd	nd
*IBSP*	nd	nd
*KRT15*	nd	nd
*ADIPOQ*	37.59 ± 0.01	4.59
*AAK1*	29.27 ± 0.06	−3.73
*KRT19*	39.33 ± 0.01	6.33

**Table 3:** rHFSC secretome analysis with mean concentration values for each biomolecule in P3 and P5 (mean ± SEM).

Biomolecule	Mean ± SEM (P3)	Mean ± SEM (P5)
EGF	0.16 ± 0.03	0.18 ± 0.04
Eotaxin	0.16 ± 0.00	0.00 ± 0.00
Fractalkine	4.38 ± 0.29	3.06 ± 0.15
G-CSF	3.43 ± 0.32	1.09 ± 0.36
GM-CSF	35.39 ± 10.69	39.75 ± 11.55
GRO/KC/CINC-1	47.32 ± 8.64	57.64 ± 7.24
IFNγ	34.01 ± 2.26	30.67 ± 0.73
IL-1α	3.79 ± 2.27	12.83 ± 5.50
IL-1β	6.99 ± 0.86	7.27 ± 0.63
IL-2	12.21 ± 1.23	12.65 ± 0.58
IL-4	4.88 ± 1.30	2.63 ± 0.00
IL-5	24.13 ± 2.69	21.13 ± 2.46
IL-6	166.36 ± 50.22	165.62 ± 0.00
IL-10	18.37 ± 1.87	18.17 ± 0.88
IL-12p70	4.34 ± 1.80	1.42 ± 0.00
IL-13	3.39 ± 0.43	8.84 ± 0.51
IL-17A	4.77 ± 0.30	7.57 ± 1.11
IL-18	8.89 ± 1.31	5.55 ± 1.24
IP-10	2.33 ± 0.31	1.96 ± 0.38
Leptin	32.31 ± 8.97	30.49 ± 4.08
MCP-1	131.64 ± 38.26	137.21 ± 19.77
MIP-1α	1.70 ± 0.57	2.19 ± 0.39
MIP-2	32.78 ± 5.77	44.02 ± 7.97
RANTES	0.51 ± 0.03	0.50 ± 0.01
VEGF	91.75 ± 3.22	98.88 ± 1.06
TGF-β1	5.82 ± 1.07	7.14 ± 1.02
TGF-β2	0.97 ± 0.04	0.98 ± 0.00
TGF-β3	0.19 ± 0.00	0.00 ± 0.00

## Data Availability

Further data on the reported results are available from the corresponding author on request.

## Abbreviations

ACTB	Beta-Actin
CFU	Colony Forming Units
DGAV	Veterinary Authorities of Portugal
DMSO	Dimethyl Sulfoxide
EGF	Epidermal Growth Factor Recombinant Protein
FBS	Bovine Fetal Serum
GAPDH	Glyceraldehyde 3-Phosphate Dehydrogenase
G-CSF	Granulocyte Colony-Stimulating Factor
GM-CSF	Granulocyte-Macrophage Colony-Stimulating Factor
GRO/KC/CINC-1	Human Growth-Regulated Oncogene/Keratinocyte Chemoattractant/Cytokine-Induced Neutrophil Chemoattractant-1
HFSCs	Hair Follicle Stem Cells
ICBAS	Abel Salazar Institute for Biomedical Sciences
IFNϒ	Interferon Gamma
IL	Interleukin
IP-10	Interferon-Gamma Inducible Protein
MCP-1	Monocyte Chemoattractant Protein-1
MIP	Macrophage Inflammatory Protein
ORBEA	Organism Responsible for Animal Welfare
PDT	Population Doubling Time
RANTES	Regulated upon Activation, Normal T Cell Expressed and Presumably Secreted
rHFSCs	Rat Hair Follicle Stem Cells
RT-PCR	Reverse Transcriptase Polymerase Chain Reaction
SEM	Standard Error of the Mean
TGFβ	Transforming Growth Factor Beta
TNFα	Tumor Necrosis Factor-Alpha
UP	University of Porto
VEGF	Vascular Endothelial Growth Factor
